# Astragaloside IV Protects 6-Hydroxydopamine-Induced SH-SY5Y Cell Model of Parkinson’s Disease via Activating the JAK2/STAT3 Pathway

**DOI:** 10.3389/fnins.2021.631501

**Published:** 2021-03-23

**Authors:** ZhengHu Xu, Dongfeng Yang, Xiaojing Huang, Huai Huang

**Affiliations:** ^1^Department of Neurosurgery, Hebei PetroChina Central Hospital, Langfang, China; ^2^Department of Neurology, Hebei PetroChina Central Hospital, Langfang, China

**Keywords:** Parkinson’s disease, astragaloside IV, JAK2/STAT3 pathway, SH-SY5Y cells, inflammation, oxidative stress, apoptosis, cell viability

## Abstract

**Objectives:**

Astragaloside IV (AS-IV), the main active component of Astragalus membranaceus, bears anti-inflammatory, antioxidant, and neuroprotective activity. Parkinson’s disease (PD) is a common neurodegenerative disease. This study explored the protective effect of AS-IV on the cell model of PD.

**Materials and Methods:**

SH-SY5Y cells were incubated with different concentrations (10, 50, 100, 150, and 200 μM) of 6-hydroxydopamine (6-OHDA) for 0, 3, 6, 12, 24, and 48 h to establish the PD cell model. Different concentrations (0, 25, 50, 100, 150, and 200 μM) of AS-IV or 15 mM JAK2/STAT3 pathway inhibitor SC99 was added for intervention 2 h before 6-OHDA treatment. The viability and morphological damage of 6-OHDA-treated SH-SY5Y cells were measured using MTT assay and Hoechst 33258 staining. The expression of microtubule associated protein 2 (MAP2) was detected by immunofluorescence staining. The levels of inflammation and oxidative stress were measured using ELISA. Apoptosis of 6-OHDA-treated SH-SY5Y cells was detected using flow cytometry, and phosphorylation level of JAK2 and STAT3 were detected using Western blot analysis.

**Results:**

The survival rate of SH-SY5Y cells treated with 100 μM 6-OHDA for 24 h was about 50%. AS-IV (25–100 μM) significantly improved the viability (all *p* < 0.01), increased MAP2 expression, and repaired the morphological damage induced by 6-OHDA. AS-IV inhibited IL-1β, IL-6, and TNF-α level (all *p* < 0.05), reduced MDA and ROS content and increased SOD concentration, thereby reducing inflammation and oxidative stress (all *p* < 0.01) in 6-OHDA-treated SH-SY5Y cells. Moreover, AS-IV decreased apoptosis rate and Bax/Bcl-2 ratio induced by 6-OHDA (all *p* < 0.05). Mechanically, AS-IV significantly increased the phosphorylation of JAK2 and STAT3 (*p* < 0.01); the addition of SC99 decreased the cell viability, increased the apoptosis rate, enhanced the levels of inflammatory factors and oxidative stress.

**Conclusion:**

AS-IV enhanced the cell viability, and inhibited apoptosis, inflammation and oxidative stress of 6-OHDA-treated SH-SY5Y cells via activating the JAK2/STAT3 signaling pathway. This study may confer novel insights for the management of PD.

## Introduction

Parkinson’s disease (PD) is a neurodegenerative disease caused by multiple factors with the incidence rate rising steadily with age, which brings increasing economic burden to the society (Ascherio and Schwarzschild, 2016). The main clinical feature of PD represents progressive impairment of voluntary movement control (Frisardi et al., 2016). Impairment of voluntary movement control can result in dyskinesia, bradykinesia and tremor, often accompanied by gait disorders, worse balance and coordination (Mazzoni et al., 2012). PD presents the pathological processes such as oxidative stress and neuroinflammation, which are aggravated by aging (Soderbom and Zeng, 2020). At present, the medical management of patients with PD is still challenging due to the limited choice of drugs (Radhakrishnan and Goyal, 2018). Intriguingly, Chinese medicinal plants have received extensive attention, and many adjuvant drugs that can inhibit or delay the neurodegenerative process have been extracted (Huang et al., 2019). Most of the current studies on PD adopt neuronal cell models, especially the SH-SY5Y lineage of neuroblastoma because human dopaminergic neurons are difficult to acquire and sustain as primary cells (Xicoy et al., 2017). Therefore, this study aimed to identify the protective role of Chinese medicinal plants in the SH-SY5Y cell model of PD.

Traditional Chinese herb medicine has the neuroprotective potential to delay the progress of PD and can treat both motor and non-motor symptoms of PD simultaneously (Zeng, 2017). Astragalus membranaceus bears extensive pharmacological actions as a primary Chinese herbal medicine, which is commonly utilized in many traditional Chinese medicine preparations (Auyeung et al., 2016). Astragaloside IV (AS-IV), a kind of cycloalkane triterpene glycoside chemical, is a major active compound derived from Astragalus membranaceus (Zhang et al., 2020). The effects of AS-IV on anti-inflammation, antioxidation, and neuroprotection have been unveiled (Xia et al., 2020). The application of AS-IV notably meliorates the behavioral and neurochemical impairments, which is accepted as a promising therapeutic agent for the management of neurological diseases (Costa et al., 2019). AS-IV inhibits endoplasmic reticulum stress-mediated neuronal apoptosis in a murine model of PD (Ge et al., 2020). AS-IV pretreatment dose-dependently attenuates the 6-hydroxydopamine (6-OHDA)-induced loss of the dopaminergic neurons (Chan et al., 2009). AS-IV protects against MPP^+^-induced dopaminergic neurotoxicity in SH-SY5Y cells via inhibiting the Bax-mediated pathway (Zhang et al., 2012). These previous findings have indicated that AS-IV may be a promising neuroprotective agent for PD, so this study aims to explore more possible protective mechanisms of AS-IV in a cell model of PD.

Age-related degeneration in the JAK2/STAT3 axis exerts crucial effect on the pathogenesis of neurodegenerative diseases (Chiba et al., 2009). JAK2/STAT3 pathway is implicated in the neuroprotective effect of linagliptin on behavior disorders in mice (Elbaz et al., 2018). Bisdemethoxycurcumin is reported to play a protective role in a rotenone-induced PD model via the JAK2/STAT3 signaling pathway (He et al., 2020). However, whether AS-IV can prevent the injury of 6-OHDA-treated SH-SY5Y cells via regulating the JAK2/STAT3 pathway remains unclear. Therefore, we determined the role of the JAK2/STAT3 pathway in the mechanism of AS-IV protecting PD cells, which could provide a new theoretical basis for the neuroprotective effects of AS-IV and contribute to design effective drugs for PD in the future.

## Materials and Methods

### SH-SY5Y Cell Culture and Treatment

SH-SY5Y cells purchased from BeNa Culture Collection (Suzhou, Jiangsu, China) were seeded in culture flasks containing complete medium [90% basic Dulbecco’s modified Eagle’s medium (DMEM, Invitrogen, Carlsbad, CA, United States), 10% heat inactivated fetal bovine serum, 100 μg/mL streptomycin and 100 U/mL penicillin (Sangon Biotech Co., Ltd, Shanghai, China)] at 37°C and 5% CO_2_. The medium was refreshed every other day and cells were passaged every 2–3 days. The cells at passage 3 were used for the subsequent experiments.

SH-SY5Y cells in good conditions were treated with different concentrations (10, 50, 100, 150, and 200 μM) of 6-OHDA (Sigma-Aldrich, Merck KGaA, Darmstadt, Germany) for different time (0, 3, 6, 12, 24, and 48 h) (Chen et al., 2020). The concentration and time of 6-OHDA intervention at 50% cell activity were selected as the experimental conditions. Different concentrations (25, 50, 100, 150, and 200 μM) of AS-IV (National Institute for the Control of Pharmaceutical and Biological Products, Beijing, China) was added to the medium 2 h before 6-OHDA treatment, or 15 μL JAK2/STAT3 pathway inhibitor SC99 [2-(2-(3-chloro-4-fluorophenyl)hydrazono)-3-(4-chlorophenyl)-3-oxo-propanenitrile, 2-(2-(2-(3-4-fluorophenyl)hydrazine)-3-(4-chlorophenyl)-3-oxo-acrylonitrile)] (15 mM, Sigma-Aldrich) was added to interfere with the pathway (Zhang et al., 2016).

### 3-(4,5-Dimethylthiazol-2-yl)-2,5-Diphenyltetrazolium Bromide (MTT) Assay

The cells at passage 3 in logarithmic growth phase were made into single cell suspension, and then seeded into the 96-well plate (1 × 10^5^ cells/well). After 24 h of culture, the cells showed a good adherence ability and growth state. The original medium was replaced by the medium free of penicillin-streptomycin solution. Then, 200 μL MTT 500 μg/mL (C009S, Beyotime, Shanghai, China) was supplemented to each well for another 4 h of incubation at 37°C. Afterward, the culture solution was sucked away and 200 μL dimethyl sulphoxide (DMSO) solution was added. The plate was shaken for 10 min to dissolve the crystal completely. The optical density (OD) of each well was measured at a wavelength of 492 nm on a microplate reader.

### Immunofluorescence Staining

SH-SY5Y cells were fixed in 4% paraformaldehyde for 10 min and then washed with PBS again. Next, the cells were incubated with the primary antibody mouse anti-MAP2 (M1406, 1:500, Sigma-Aldrich) at 4°C overnight. After washing with PBS, the cells were incubated with goat anti-mouse IgG H&L (Alexa Fluor^®^ 647) (ab150115, Abcam) for 1 h at room temperature, and then observed under Zeiss LSM700 confocal microscope (Carl Zeiss, Germany).

### Hoechst 33258 Staining

SH-SY5Y cells in logarithmic growth phase were seeded in the 24-well plate (1 × 10^5^ cells/well). The cells were cultured with 6-OHDA for 24 h upon cell confluence reaching 70–80%. Then, SH-SY5Y cells were rinsed with phosphate-buffered saline (PBS) three times at 4°C and fixed in 4% paraformaldehyde for 10 min, following PBS washing again. Next, cells were stained with 500 μL Hoechst 33258 (C1018, Beyotime) for 10 min. Following PBS washing, cells were observed under a fluorescence microscope (FV1000, Olympus Optical Co., Ltd, Tokyo, Japan).

### Enzyme-Linked Immunosorbent Assay (ELISA)

The levels of interleukin (IL)-1β, IL-6, and tumor necrosis factor (TNF)-α in cells were measured using the ELISA kits (R&D systems, Minneapolis, MN, United States).

### Detection of Oxidative Stress

The content of Malondialdehyde (MDA) in cells was detected using the MDA kit (ab118970, Abcam, Cambridge, MA, United States). The level of reactive oxygen species (ROS) was detected using the ROS/Superoxide kit (ab139476, Abcam). The content of superoxide dismutase (SOD) was detected using the SOD kit (ab65354, Abcam).

### Flow Cytometry

The cells were mildly trypsinized, washed with PBS and stained with annexin V-FITC and propidium iodide (PI) (V13242, Thermo Fisher Scientific Inc., Waltham, MA, United States). The apoptosis rate was detected using the flow cytometer (MoFloAstrios EQ, Beckman Coulter Inc, CA, United States), among which Q2 was late apoptotic cell, Q4 was early apoptotic cell, Q3 was living cell, and Q1 was dead cell. The apoptotic rate = (Q2 + Q4)/(Q1 + Q2 + Q3 + Q4) × 100%.

### Western Blot Analysis

Total protein of cells was extracted in radio-immunoprecipitation assay buffer (Beyotime) containing protease inhibitors. The concentration of protein extracted from cells was tested using the bicinchoninic acid assay kit (Beyotime). The protein was denatured at 100°C for 5 min. Then, the protein was separated on sodium dodecyl sulfate-polyacrylamide gel electrophoresis and transferred onto polyvinylidene difluoride membranes (Millipore, Bedford, MA, United States). The membranes were blocked in buffer at 4°C for 2 h and cultured with the primary antibodies at 4°C overnight: Bax (1:1000, ab32503, Abcam), Bcl-2 (1:1000, ab32124, Abcam), JAK2 (1:5000, ab108596, Abcam), p-JAK2 (1:1000, ab32101, Abcam), STAT3 (1:2000, ab68153, Abcam), p-STAT3 (1:2000, ab76315, Abcam), and (β-actin (1:1000, ab8226, Abcam). Afterward, the membranes were washed with tris-buffered saline (TBS) and Tween-20 (TBST) three times and cultured with the secondary antibody mouse anti-rabbit immunoglobulin G (IgG) (1:2000, ab6721, Abcam) at 4°C for 4 h. Subsequently, the membranes were developed and visualized using the enhanced chemiluminescence reagent (Thermo Fisher Scientific Pierce, Rockford, IL, United States). The protein band was observed with β-actin acting as the internal reference. The gray value of the target band was analyzed by Image Lab software (National Institutes of Health, Maryland, United States).

### Statistical Analysis

SPSS 21.0 (IBM Corp., Armonk, NY, United States) was utilized for data analysis. Kolmogorov-Smirnov test showed that the data were in normal distribution and expressed as mean ± standard deviation. The *t* test was adopted for analysis of comparisons between two groups. The one-way analysis of variance (ANOVA) was applied for comparisons among multi-groups. Dunnett’s multiple comparisons test or Tukey’s multiple comparison test was employed for the *post hoc* test after ANOVA. The *p* value was obtained from a two-tailed test, and *p* < 0.05 meant a statistical difference and *p* < 0.01 indicated a very significant difference.

## Results

### Selection of Suitable Concentration and Time for 6-OHDA Treatment of Cells

**FIGURE 1:**
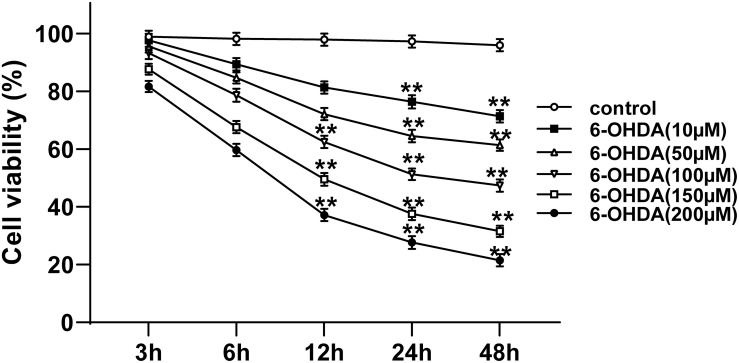
Selection of suitable concentration and time for 6-OHDA treatment of cells. The cell viability of SH-SY5Y cells treated with different concentrations (10, 50, 100, 150, and 200 μM) of 6-OHDA at 0, 3, 6, 12, 24, and 48 h was detected using MTT assay. Each experiment was repeated for three times independently. Data are presented as mean ± standard deviation. One-way ANOVA was employed for the comparisons among multiple groups and Dunnett’s multiple comparisons test was applied for the *post hoc* test, ^∗∗^*p* < 0.01 vs. control group at the same time point.

The 6-OHDA-treated SH-SY5Y cells were used to establish the cell model of PD. The viability of 6-OHDA-treated SH-SY5Y cells at different time points and concentrations was measured using MTT assay. The viability of 6-OHDA-treated SH-SY5Y cells was decreased rapidly in the first 24 h, and then slowed down after 24 h; the cell viability showed a dose-dependent manner. The viability of cells incubated with 100 μM 6-OHDA for 24 h was nearly 50% (all *p* < 0.01; Figure 1). Therefore, 100 μM and 24 h were used as the concentration and time condition of subsequent experiments.

### AS-IV Improved SH-SY5Y Cell Viability and Repaired Morphological Damage Induced by 6-OHDA

**FIGURE 2:**
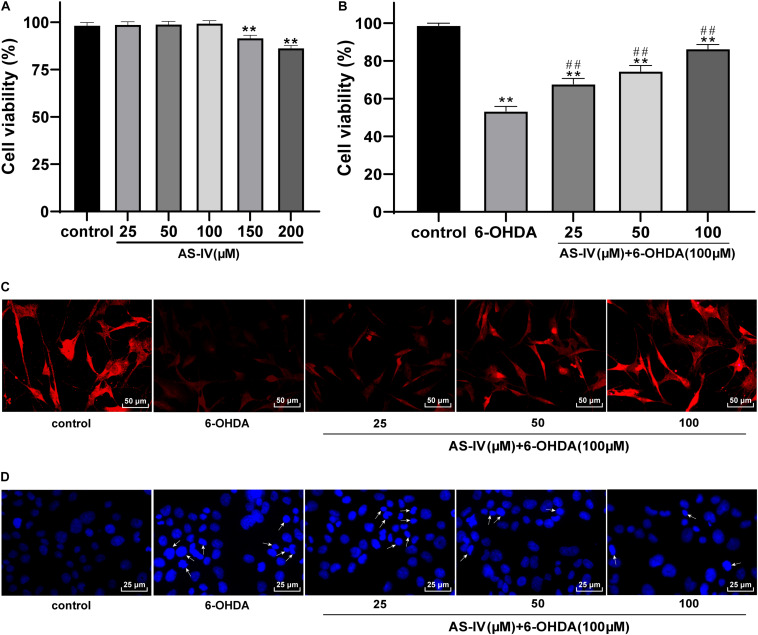
AS-IV improved SH-SY5Y cell viability and repaired morphological damage induced by 6-OHDA. **(A)** Effect of different concentrations of AS-IV (25, 50, 100, 150, and 200 μM) on the SH-SY5Y cell viability were detected using MTT assay. **(B)** Protective effect of AS-IV (25, 50, and 100 μM) on SH-SY5Y cells treated with 6-OHDA (100 μM) was observed using MTT assay. **(C)** The expression of MAP2 was detected using immunofluorescence staining. **(D)** Effect of AS-IV (25, 50, and 100 μM) on morphology of SH-SY5Y cells treated with 6-OHDA (100 μM) was detected using Hoechst 33258 staining; the arrowhead indicated the different situations of cell morphological damage, including the reduction and roundness of cell morphology, and the dense fluorescent granules. Each experiment was repeated for three times independently. Data are presented as mean ± standard deviation. One-way ANOVA was employed for the comparisons among multiple groups and Tukey’s multiple comparison test was applied for the *post hoc* test. **p < 0.01 vs. control group; ##*p* < 0.01 vs. 6-OHDA groups.

AS-IV has been proven to have a cytoprotective function (Chan et al., 2009; Yang et al., 2020), while the role of AS-IV in PD remains unclear. MTT assay was adopted to evaluate the effect of AS-IV on SH-SY5Y cell viability. We found that 25, 50, and 100 μM AS-IV did not affect the SH-SY5Y cell viability, and the cell viability began to decline till the concentration of AS-IV reached 150 μM, which significantly differed from that of the control group (all *p* < 0.01; Figure 2A), indicating that 150 μM AS-IV had certain toxic and side effects on SH-SY5Y cells. Then, 6-OHDA-treated SH-SY5Y cells were incubated with 25, 50, and 100 μM AS-IV, and we revealed that AS-IV could significantly improve the survival rate of cells in a dose-dependent manner (all *p* < 0.01; Figure 2B). We detected the expression of neuron dendrite marker MAP2 by immunofluorescence staining (Wang Y. L. et al., 2020), and the results demonstrated that the expression of MAP2 in 6-OHDA-treated group was decreased, while AS-IV could enhance the expression of MAP2 in varying degrees (Figure 2C). Hoechst 33258 staining was used to observe the effect of AS-IV on the morphological damage of cells treated with 6-OHDA. The 6-OHDA-treated SH-SY5Y cells showed severe damage, suspension, shrinkage and dense particle fluorescence, while AS-IV could repair the cell morphology damage (Figure 2D). These results indicated that AS-IV improved the viability and repaired the morphological damage of 6-OHDA-treated SH-SY5Y cells.

### AS-IV Inhibited Levels of Inflammatory Factors and Oxidative Stress in 6-OHDA-Treated SH-SY5Y Cells

**FIGURE 3:**
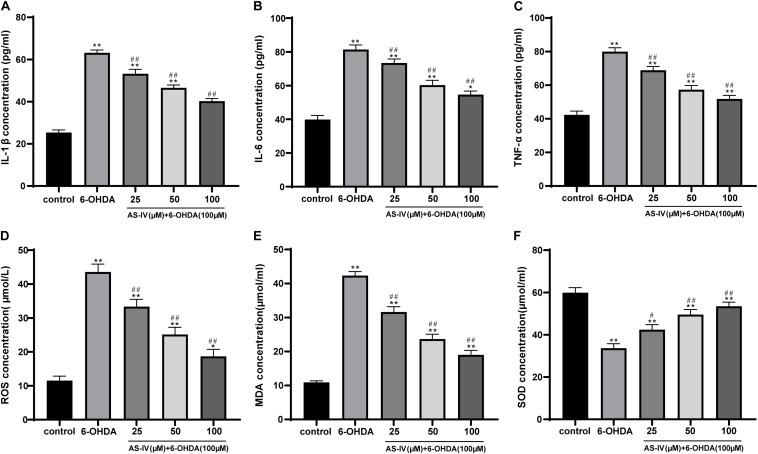
AS-IV inhibited the levels of inflammatory factors and oxidative stress in 6-OHDA-treated SH-SY5Y cells. SH-SY5Y cells were treated with different concentrations of AS-IV (25, 50, and 100 μM) for 2 h, and then treated with 6-OHDA (100 μM) for 24 h. **(A–C)** Levels of IL-1β, IL-6, and TNF-α in 6-OHDA-treated SH-SY5Y cells were detected using the ELISA kits. **(D–F)** Changes of MDA, ROS, and SOD level in 6-OHDA-treated SH-SY5Y cells were detected using corresponding kits. Each experiment was repeated for three times independently. Data are presented as mean ± standard deviation. One-way ANOVA was employed for the comparisons among multiple groups and Tukey’s multiple comparison test was applied for the *post hoc* test, ^∗^*p* < 0.05, ^∗∗^*p* < 0.01 vs. control group; ^#^*p* < 0.05, ^##^*p* < 0.01 vs. 6-OHDA groups.

It has been reported that inflammatory reaction and oxidative stress injury may occur in 6-OHDA-treated cells (Pignataro et al., 2017). We detected the levels of inflammatory factors in SH-SY5Y cells using the ELISA kits and found that different doses of AS-IV could significantly inhibit the levels of IL-1β, IL-6, and TNF-α in 6-OHDA-treated SH-SY5Y cells relative to that of the control cells (all *p* < 0.05; Figures 3A–C). Moreover, we detected the changes of oxidative stress related indexes and found that 6-OHDA induction resulted in increased MDA and ROS but decreased SOD in SH-SY5Y cells, while AS-IV could effectively reverse the changes of these oxidative damage indexes (all *p* < 0.05; Figures 3D–F). It was implied that AS-IV inhibited inflammatory responses of 6-OHDA-treated SH-SY5Y cells and protected cells against oxidative stress.

### AS-IV Inhibited Apoptosis of 6-OHDA-Treated SH-SY5Y Cells and Decreased Bax/Bcl-2

**FIGURE 4:**
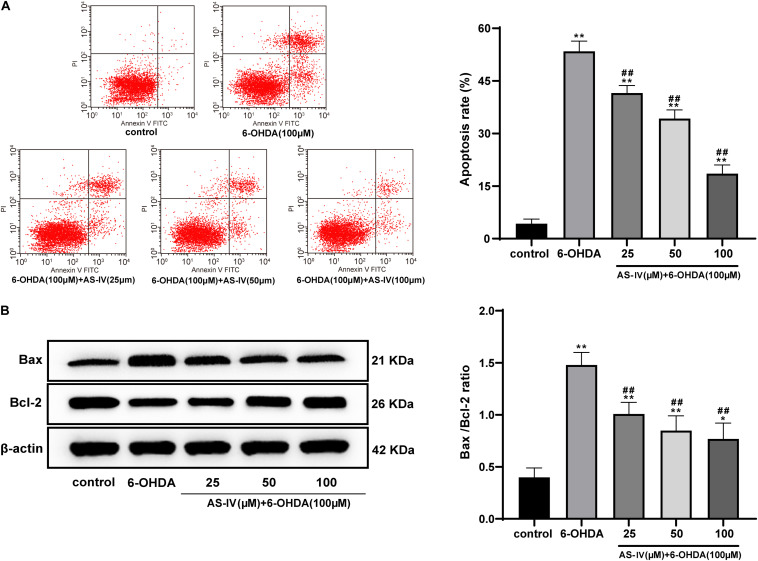
AS-IV inhibited the apoptosis of 6-OHDA-treated SH-SY5Y cells and decreased Bax/Bcl-2. **(A)** Apoptosis rate of 6-OHDA-treated SH-SY5Y cells was detected using flow cytometry. **(B)** Levels of Bax and Bcl-2 were detected using Western blot analysis. Each experiment was repeated for three times independently. Data are presented as mean ± standard deviation. One-way ANOVA was employed for the comparisons among multiple groups and Tukey’s multiple comparison test was applied for the *post hoc* test, ^∗∗^*p* < 0.01 vs. control group; ^#^*p* < 0.05, ^##^*p* < 0.01 vs. 6-OHDA groups.

Emerging evidences have shown that 6-OHDA can induce apoptosis and promote the levels of apoptotic proteins such as Bax (Wang T. et al., 2020). Hence, we explored the role of AS-IV in the apoptosis of 6-OHDA-treated SH-SY5Y cells. The apoptosis rate of cells in the 6-OHDA treatment group was notably increased relative to that in the control group, and AS-IV treatment decreased the apoptosis rate of 6-OHDA-treated SH-SY5Y cells to varying degrees (all *p* < 0.01; Figure 4A), suggesting that AS-IV could inhibit the apoptosis of cells treated with 6-OHDA. The levels of Bax and Bcl-2 were further detected using Western blot analysis, and we found that AS-IV treatment decreased the level of Bax and increased the level of Bcl-2, showing reduced Bax/Bcl-2 (all *p* < 0.05; Figure 4B).

### AS-IV Activated the JAK2/STAT3 Pathway in 6-OHDA-Treated SH-SY5Y Cells

**FIGURE 5:**
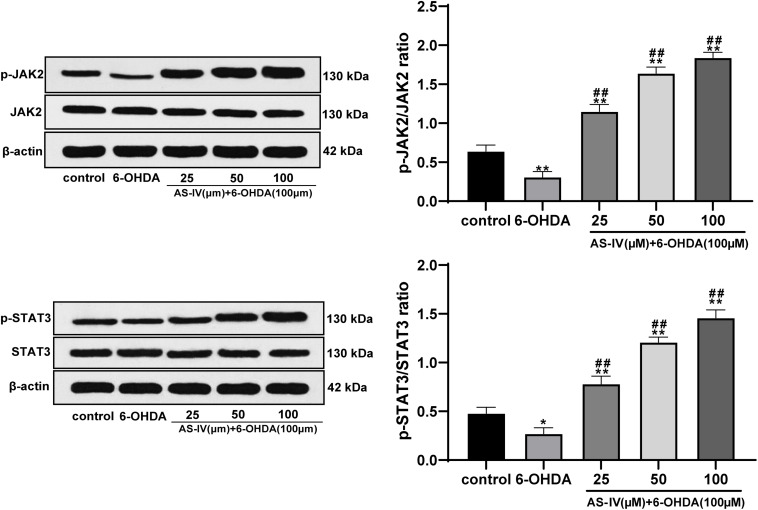
AS-IV activated the JAK2/STAT3 pathway in 6-OHDA-treated SH-SY5Y cells. Levels of JAK2, p-JAK2, STAT3, and p-STAT3 were detected using Western blot analysis. Each experiment was repeated for three times independently. Data are presented as mean ± standard deviation. One-way ANOVA was employed for the comparisons among multiple groups and Tukey’s multiple comparison test was applied for the *post hoc* test, ^∗^*p* < 0.05, ^∗∗^*p* < 0.01 vs. control group; ^##^*p* < 0.01 vs. 6-OHDA groups.

Previous literature has revealed that AS-IV can regulate the JAK2/STAT3 pathway (Xu et al., 2020). We speculated that AS-IV could regulate PD cells through the JAK2/STAT3 pathway. Western blot analysis was adopted to detect the levels of JAK2, p-JAK2, STAT3, and p-STAT3, and we found that AS-IV treatment notably increased the phosphorylation levels of JAK2 and STAT3 in 6-OHDA-treated SH-SY5Y cells (all *p* < 0.01; Figure 5). AS-IV played a protective role in 6-OHDA-treated SH-SY5Y cells by activating the JAK2/STAT3 pathway.

### AS-IV Protected 6-OHDA-Treated SH-SY5Y Cells via Activating the JAK2/STAT3 Pathway

**FIGURE 6:**
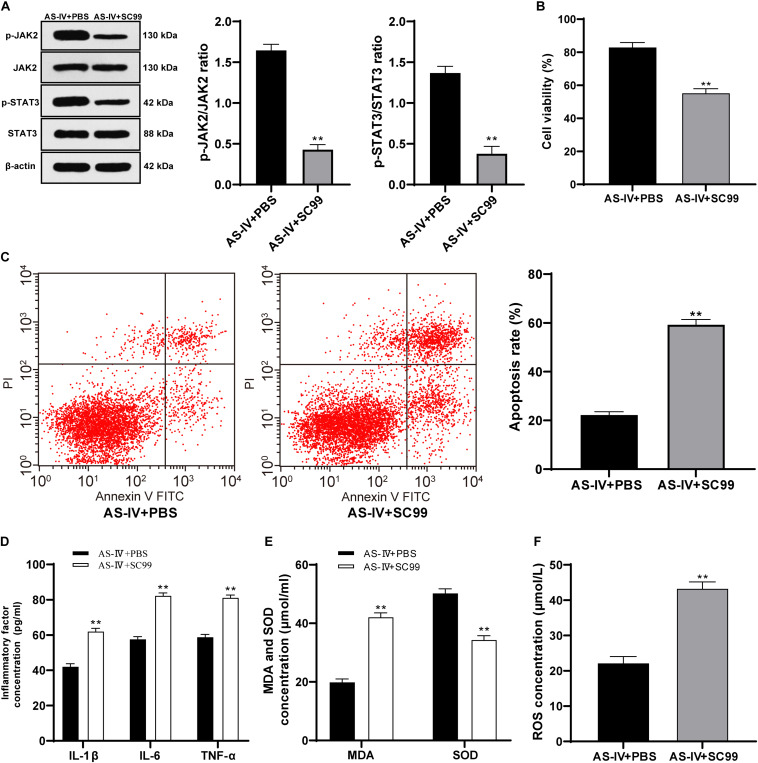
AS-IV (100 μM) protected 6-OHDA-treated SH-SY5Y cells via activating the JAK2/STAT3 pathway. The JAK2/STAT3 pathway inhibitor SC99 (15 mM) was added to the cells treated with AS-IV (100 μM), with PBS as the control. **(A)** Levels of p-JAK2/JAK2 and p-STAT3/STAT3 were detected using Western blot analysis. **(B)** Viability of 6-OHDA-treated SH-SY5Y cells was detected using MTT assay. **(C)** Apoptosis rate of 6-OHDA-treated SH-SY5Y cells was detected flow cytometry. **(C)** Levels of IL-1β, IL-6, and TNF-α in 6-OHDA-treated SH-SY5Y cells were detected using the ELISA kits. **(D)** Changes of MDA, ROS and SOD levels in 6-OHDA-treated SH-SY5Y cells were detected using corresponding kits. Each experiment was repeated for three times independently. Data are presented as mean ± standard deviation. Data in panels **(A–F)** were analyzed using *t* test, **p < 0.01 vs. AS-IV ± PBS group.

To confirm that AS-IV played a regulatory role in 6-OHDA-treated SH-SY5Y cells via activating the JAK2/STAT3 pathway, we conducted joint experiments. The JAK2/STAT3 pathway inhibitor SC99 (15 mM) was added to the cells treated with AS-IV (100 μM), with PBS as the control. The p-JAK2/JAK2 and p-STAT3/STAT3 of cells in the AS-IV + SC99 group were significantly declined compared with that in the AS-IV + PBS group (both *p* < 0.01; Figure 6A). The AS-IV + SC99 group showed reduced cell viability and a promoted apoptosis rate compared with those in the AS-IV + PBS group (both *p* < 0.01; Figures 6B,C). The levels of inflammatory factors (IL-1β, IL-6, and TNF-α) in cells in the AS-IV + SC99 group were notably promoted compared with those in the AS-IV + PBS group (all *p* < 0.01; Figure 6D). Additionally, the AS-IV + SC99 group showed increased MDA and ROS levels, but a decreased SOD level compared with those in the AS-IV + PBS group (all *p* < 0.01; Figures 6E,F). All these results confirmed that AS-IV could inhibit apoptosis and alleviate inflammation and oxidative stress of 6-OHDA-treated SH-SY5Y cells by activating the JAK2/STAT3 pathway.

## Discussion

Parkinson’s disease poses a grave threat to the health and life of the elderly, with limited therapies to alleviate clinical symptoms (Huang et al., 2020). Chinese traditional herbal has been applied in the management of senile diseases for a long time, and its pharmacological effects on improving the symptoms or intervening pathogenesis of neurodegenerative diseases have been recognized (Law et al., 2017). AS-IV extracted from Astragalus membranaceus has been proven to bear neuroprotective effects in animal models of neurodegenerative diseases (Zhao et al., 2020). This study was primarily concentrated on the protective function of AS-IV in the cell model of PD via the JAK2/STAT3 pathway.

Administration of 6-OHDA can induce types of physiological events in animal models, including neuronal death and PD like behavior disorders (Li et al., 2016). 6-OHDA is valuable in promoting the understanding of the underlying mechanisms of PD symptoms, because it can reproduce the changes in basal ganglia circuits and pharmacology in patients with PD (Thiele et al., 2012). In view of this, we established the cell model of PD by inducing SH-SY5Y cells with 6-OHDA. The viability of cells treated with 100 μM 6-OHDA for 24 h was nearly 50%. Therefore, 100 μM and 24 h were used as the concentration and time condition of our subsequent experiments.

Xia et al. (2020) have also shown that AS-IV can prevent dopaminergic degeneration in PD by restraining astrocyte aging. The 6-OHDA-treated SH-SY5Y cells were incubated with diverse concentrations of AS-IV (25, 50, and 100 μM), and it was found that AS-IV dose-dependently enhanced cell viability and repaired morphological damages. AS-IV is also demonstrated to rescue MPP^+^-induced cell viability reduction *in vitro*, implying that AS-IV may act as a promising neuroprotective agent for PD (Xia et al., 2017). PD is featured by degeneration of dopaminergic neurons in substantia nigra striatum circuit, which is bound up with subsequent chronic neuroinflammation (Wei et al., 2019). A previous meta-analysis has revealed that PD is associated with higher levels of peripheral inflammatory cytokines, such as IL-1β, IL-6, and TNF-α (Qin et al., 2016). We detected the levels of inflammatory factors in 6-OHDA-treated SH-SY5Y cells and found that AS-IV could significantly inhibit the levels of IL-1β, IL-6, and TNF-α in 6-OHDA-treated SH-SY5Y cells. The pharmacological effects, including anti-inflammatory and antioxidative properties of AS-IV have been identified before (Li et al., 2018). Oxidative stress and neuroinflammation are accepted as the key process in the pathological course of PD (Yang et al., 2019). Free radicals produced by oxidative stress response can affect the structure and function of nerve cells and eventually cause neurodegenerative diseases, including PD (Jiang et al., 2016). AS-IV has been demonstrated as an active anti-oxidant for the management of neurodegenerative diseases (Sun et al., 2014). We observed that AS-IV decreased the MDA and ROS level and increased SOD level of 6-OHDA-treated SH-SY5Y cells. Zhang et al. (2012) have implied that AS-IV notably protects SH-SY5Y cells from oxidative damage induced by MPP^+^. Consistently, Yang et al. have implied that AS-IV alleviates motor disorder and dopaminergic neuron degeneration by reducing neuroinflammation and oxidative stress in a mouse model of PD (Yang et al., 2019). Moreover, apoptosis of dopaminergic neurons is a vital contributor to disability and mortality in PD patients (Ren et al., 2019). Our study unveiled that AS-IV reduced Bax/Bcl-2 and suppressed the apoptosis of 6-OHDA-treated SH-SY5Y cells. Ge et al. have suggested that AS-IV lessens the endoplasmic reticulum stress-induced neuronal apoptosis in a PD mouse model (Ge et al., 2020). Chan et al. have revealed that AS-IV can inhibit the apoptosis of dopaminergic neurons and enhances their resistance to neurotoxin *in vitro* (Chan et al., 2009). Liu et al. (2017) have indicated that AS-IV exerts a protecitve effect by reducing the apoptotic ratio and attenuating ROS overproduction in H_2_O_2_-exposed SH-SY5Y cells. In brief, AS-IV could inhibit inflammatory responses and apoptosis of PD cells and protect 6-OHDA-treated cells against oxidative stress.

Emerging evidences have indicated the vital role of JAK2/STAT3 pathway in neuroprotection and oxidative stress damage (Duan et al., 2013; Dong et al., 2016). Blocking the JAK2/STAT3 axis in hippocampal neurons leads to cholinergic dysfunction, which results in memory impairments in patients with neurodegenerative diseases (Chiba et al., 2009). Hence, we detected the key indicator levels of the JAK2/STAT3 pathway and found that AS-IV treatment notably increased the phosphorylation levels of JAK2 and STAT3. In addition, we employed the JAK2/STAT3 pathway inhibitor SC99 for the joint experiments. He et al. have discovered a protective influence in a rotenone-induced PD model via enhancing the JAK2/STAT3 pathway (He et al., 2020). Wang et al. have also shown that AS-IV can activate the JAK2/STAT3 pathway to stimulate angiogenesis, thereby playing a role in the clinical treatment of ischemic diseases (Wang et al., 2013). All the results confirmed that AS-IV could restrain apoptosis and alleviate inflammatory and oxidative stress in 6-OHDA-treated SH-SY5Y cells via activating the JAK2/STAT3 pathway.

To sum up, we are the first to demonstrate that AS-IV exerts protective effects on 6-OHDA-treated SH-SY5Y cells via activating the JAK2/STAT3 pathway. The deeper regulatory mechanism between AS-IV and the JAK2/STAT3 pathway demands further study. In the future, we shall carry out more prospective trials to transition our findings to clinical applications.

## Data Availability Statement

The original contributions presented in the study are included in the article/Supplementary Material, further inquiries can be directed to the corresponding author.

## Author Contributions

ZX is the guarantor of integrity of the entire study and contributed to the concepts and design of this study, experimental studies, and the data acquisition and analysis. DY and XH contributed to the definition of intellectual content and literature research of this study. HH took charge of the manuscript preparation and review. All authors read and approved the final manuscript.

## Conflict of Interest

The authors declare that the research was conducted in the absence of any commercial or financial relationships that could be construed as a potential conflict of interest.
