# Supplementary material for: Astragaloside IV Protects 6-Hydroxydopamine-Induced SH-SY5Y Cell Model of Parkinson’s Disease via Activating the JAK2/STAT3 Pathway
Source: Front Neurosci. 2021 Mar 23;15:631501. doi: 10.3389/fnins.2021.631501 (PMC8021720; doi:10.3389/fnins.2021.631501)
Supplement: Supplementary file 1 [file Presentation_1.PPTX]

## Slide 1
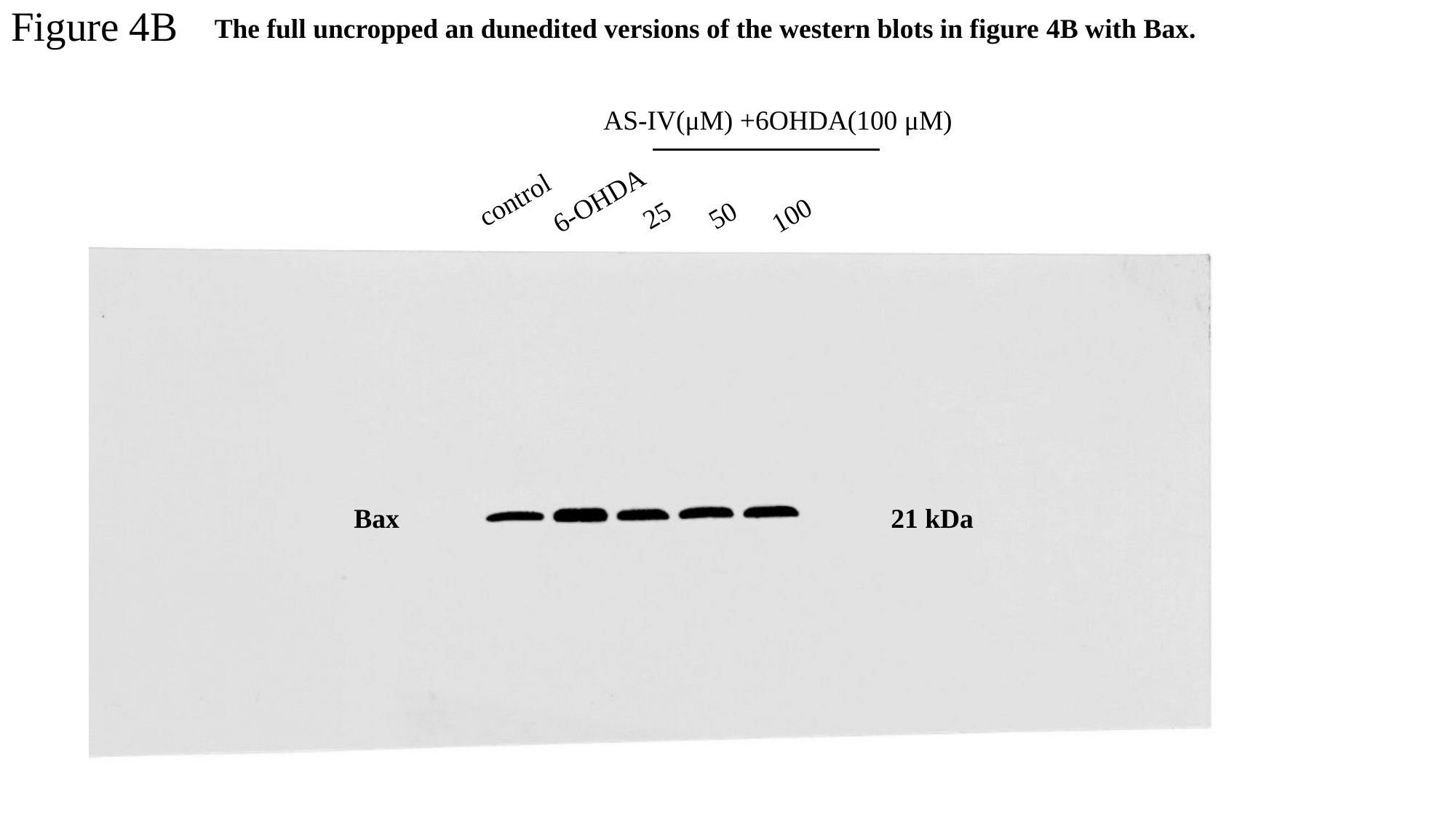

# Figure 4B
The full uncropped an dunedited versions of the western blots in figure 4B with Bax.
AS-IV(μM) +6OHDA(100 μM)
control
6-OHDA
100
50
25
21 kDa
Bax

## Slide 2
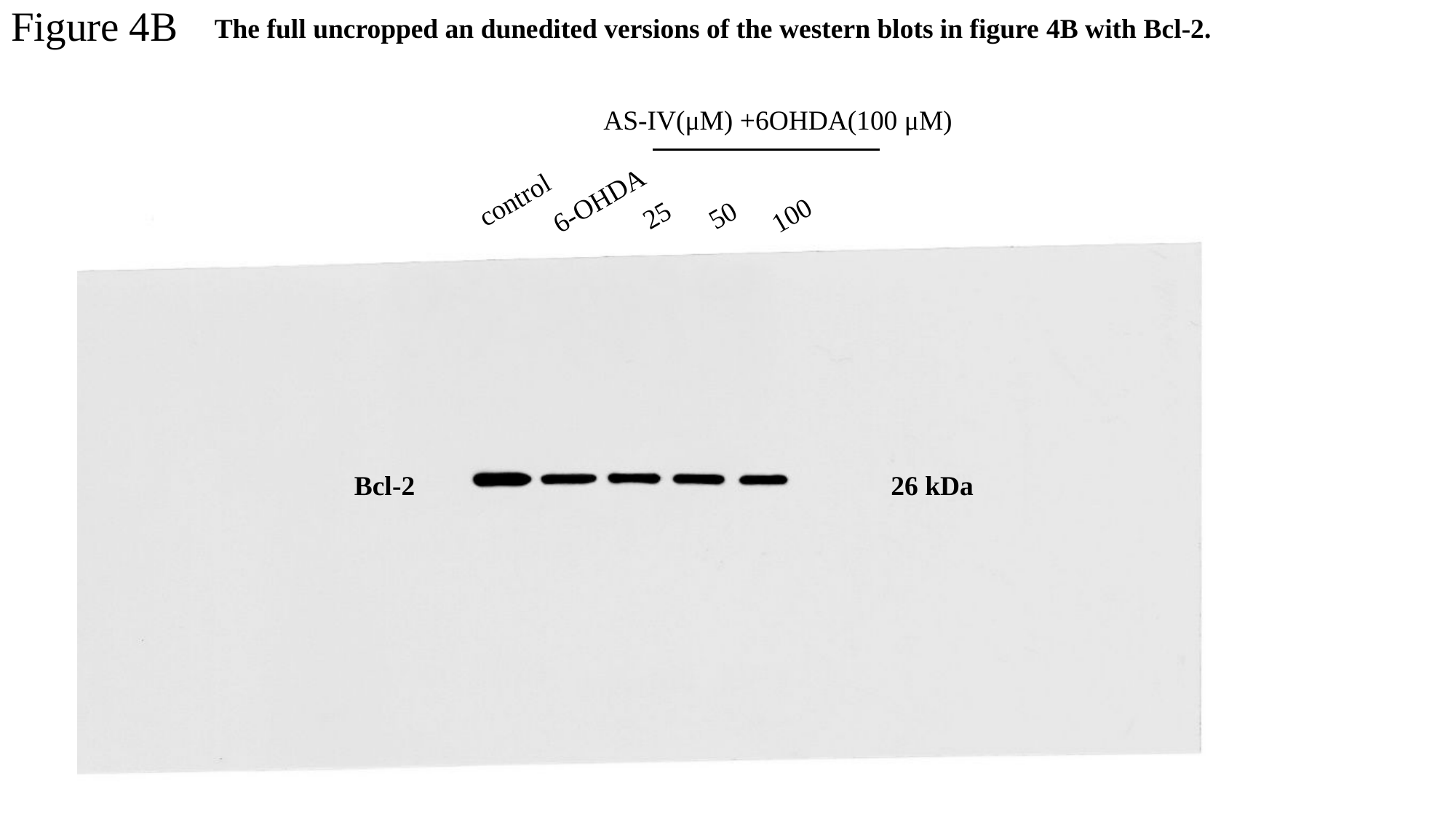

# Figure 4B
The full uncropped an dunedited versions of the western blots in figure 4B with Bcl-2.
AS-IV(μM) +6OHDA(100 μM)
control
6-OHDA
100
50
25
26 kDa
Bcl-2

## Slide 3
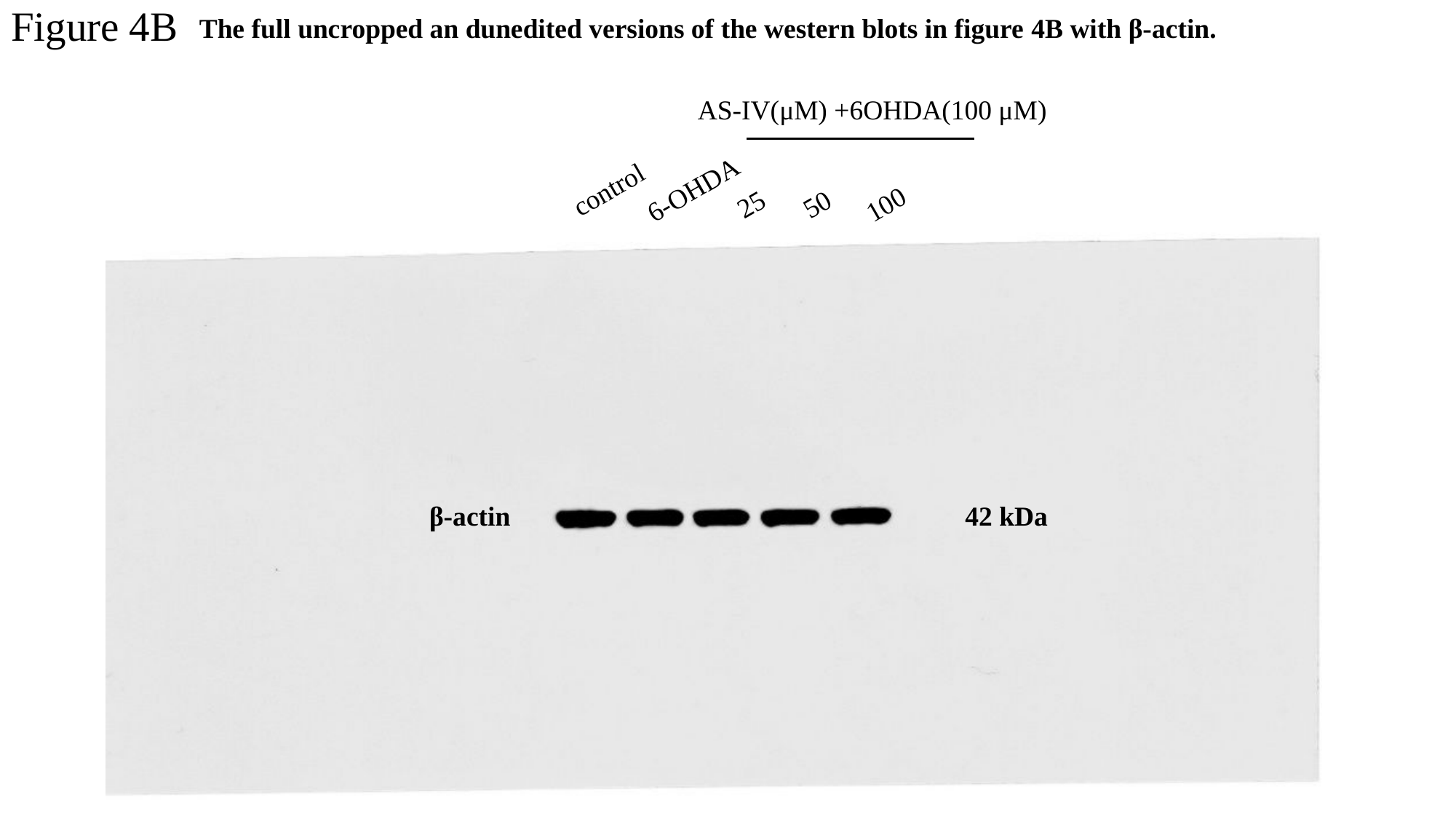

# Figure 4B
The full uncropped an dunedited versions of the western blots in figure 4B with β-actin.
AS-IV(μM) +6OHDA(100 μM)
control
6-OHDA
100
50
25
42 kDa
β-actin

## Slide 4
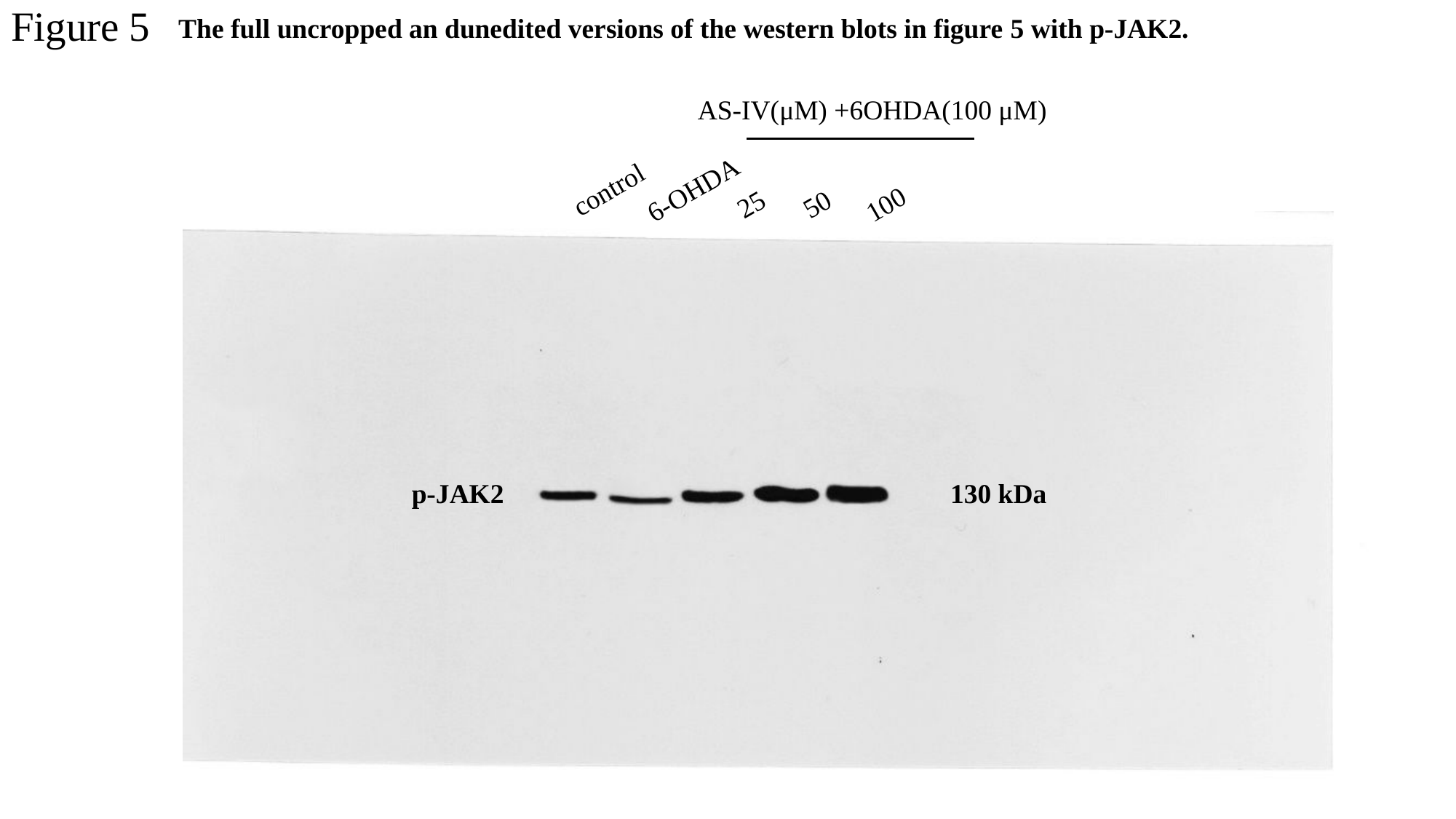

# Figure 5
The full uncropped an dunedited versions of the western blots in figure 5 with p-JAK2.
AS-IV(μM) +6OHDA(100 μM)
control
6-OHDA
100
50
25
130 kDa
p-JAK2

## Slide 5
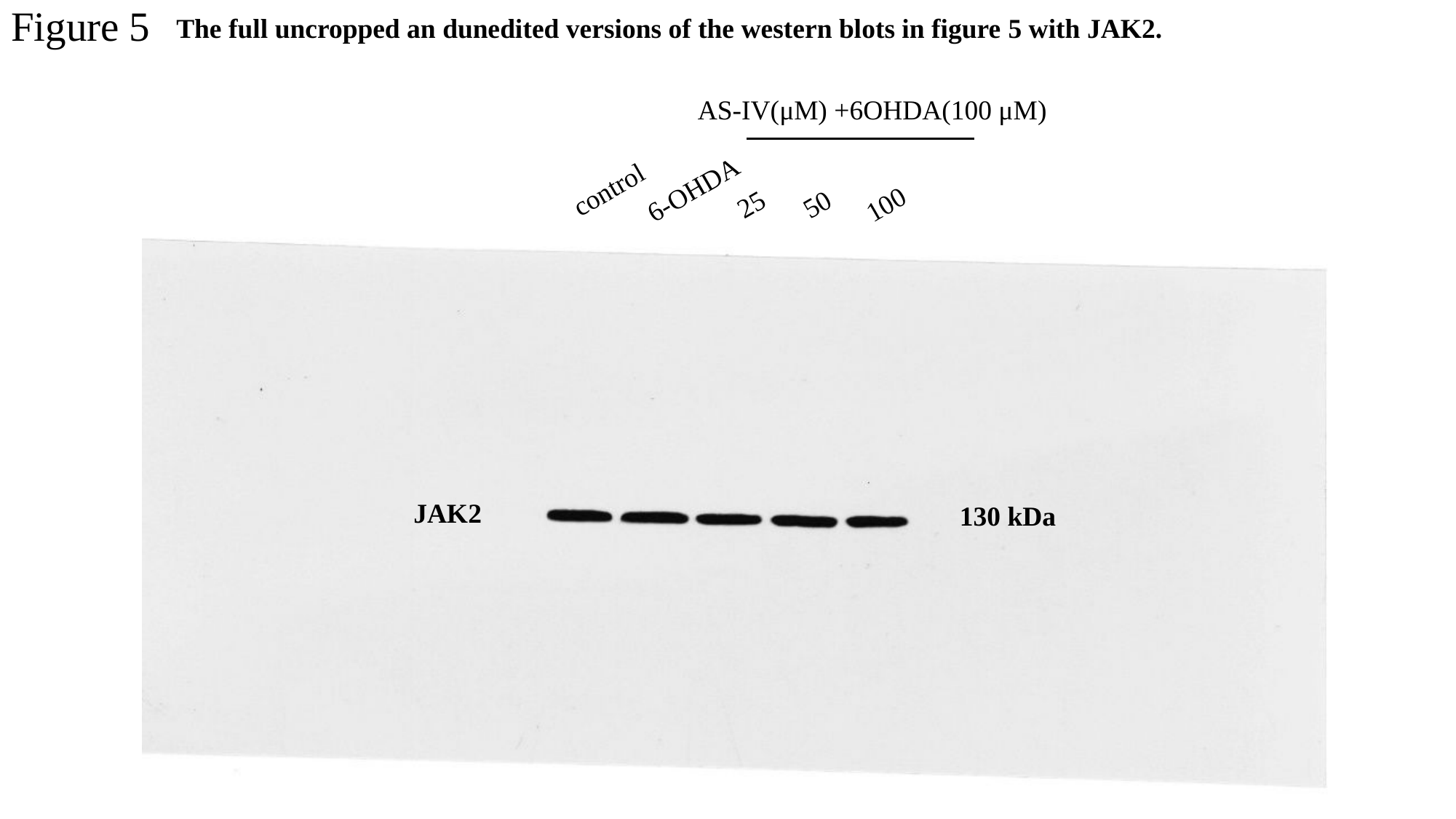

# Figure 5
The full uncropped an dunedited versions of the western blots in figure 5 with JAK2.
AS-IV(μM) +6OHDA(100 μM)
control
6-OHDA
100
50
25
JAK2
130 kDa

## Slide 6
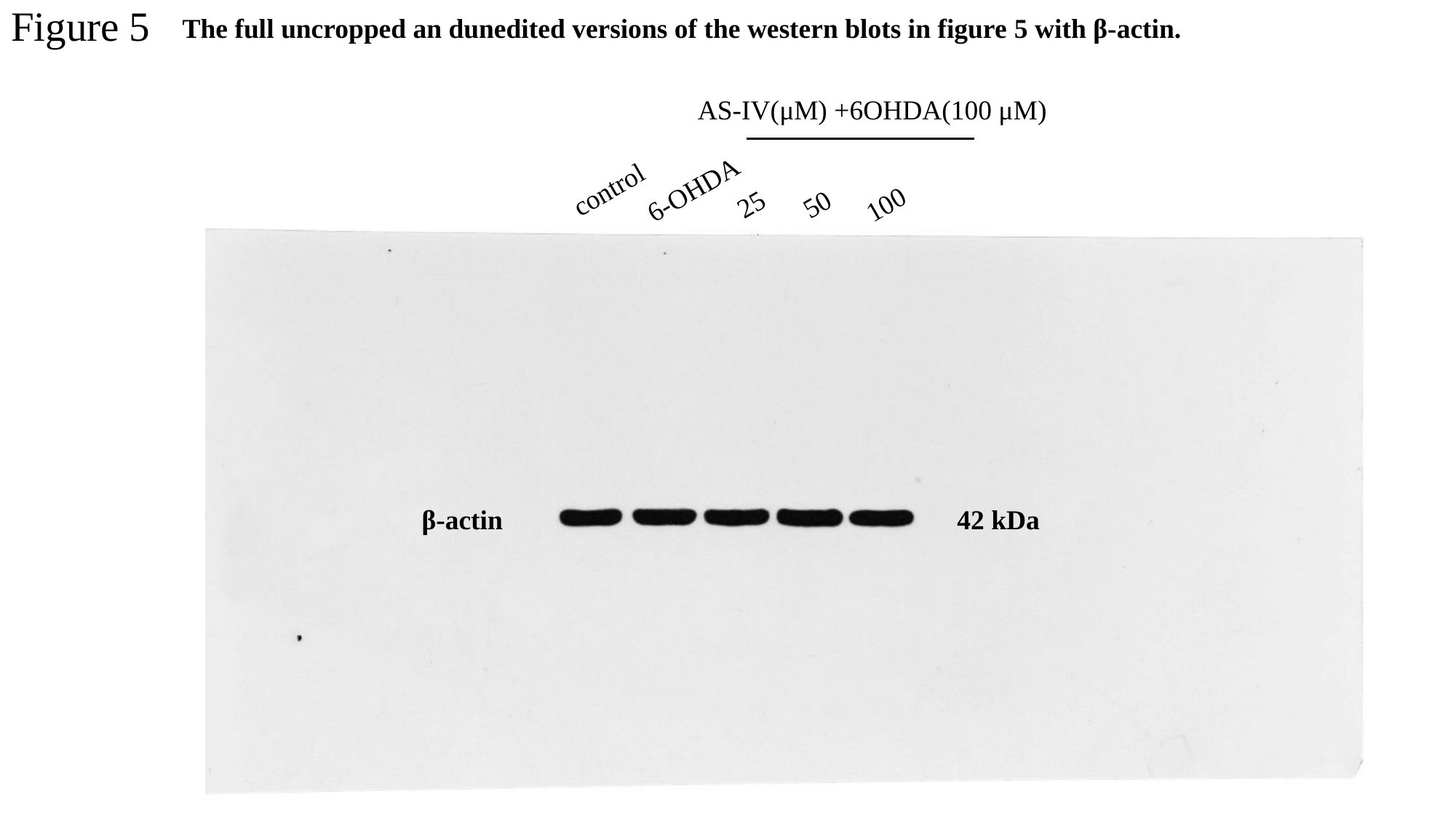

# Figure 5
The full uncropped an dunedited versions of the western blots in figure 5 with β-actin.
AS-IV(μM) +6OHDA(100 μM)
control
6-OHDA
100
50
25
42 kDa
β-actin

## Slide 7
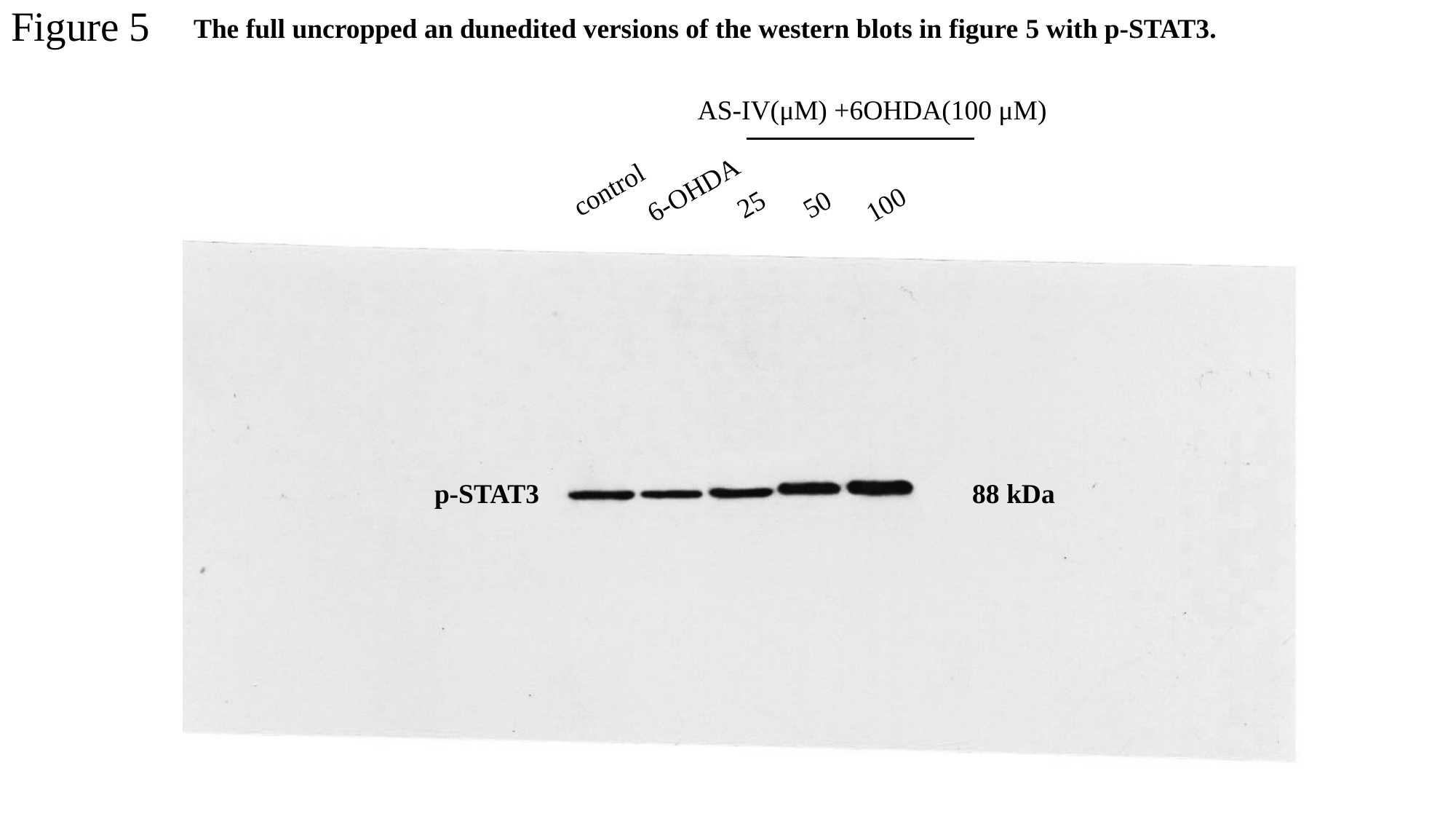

# Figure 5
The full uncropped an dunedited versions of the western blots in figure 5 with p-STAT3.
AS-IV(μM) +6OHDA(100 μM)
control
6-OHDA
100
50
25
88 kDa
p-STAT3

## Slide 8
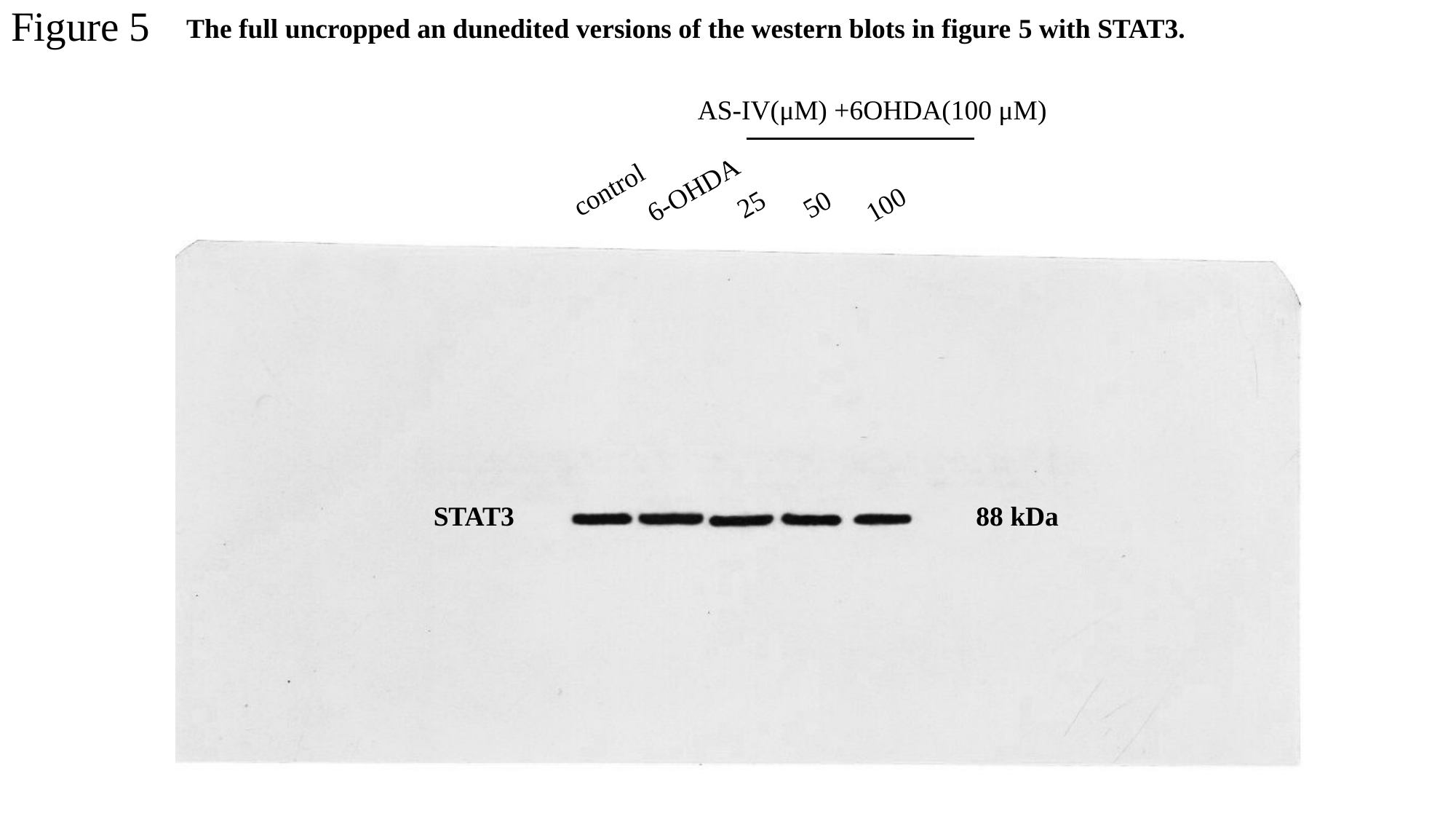

# Figure 5
The full uncropped an dunedited versions of the western blots in figure 5 with STAT3.
AS-IV(μM) +6OHDA(100 μM)
control
6-OHDA
100
50
25
88 kDa
STAT3

## Slide 9
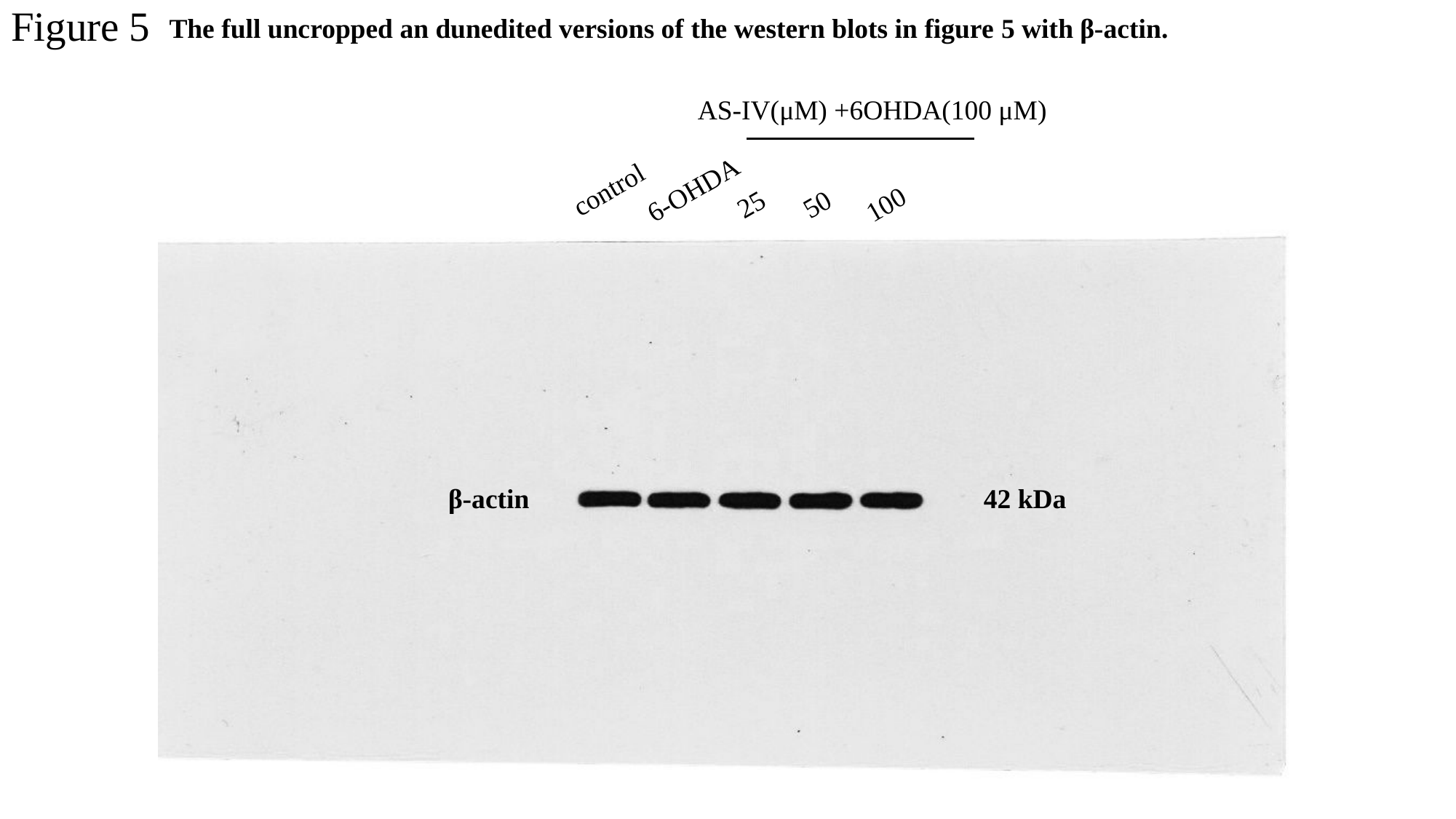

# Figure 5
The full uncropped an dunedited versions of the western blots in figure 5 with β-actin.
AS-IV(μM) +6OHDA(100 μM)
control
6-OHDA
100
50
25
42 kDa
β-actin

## Slide 10
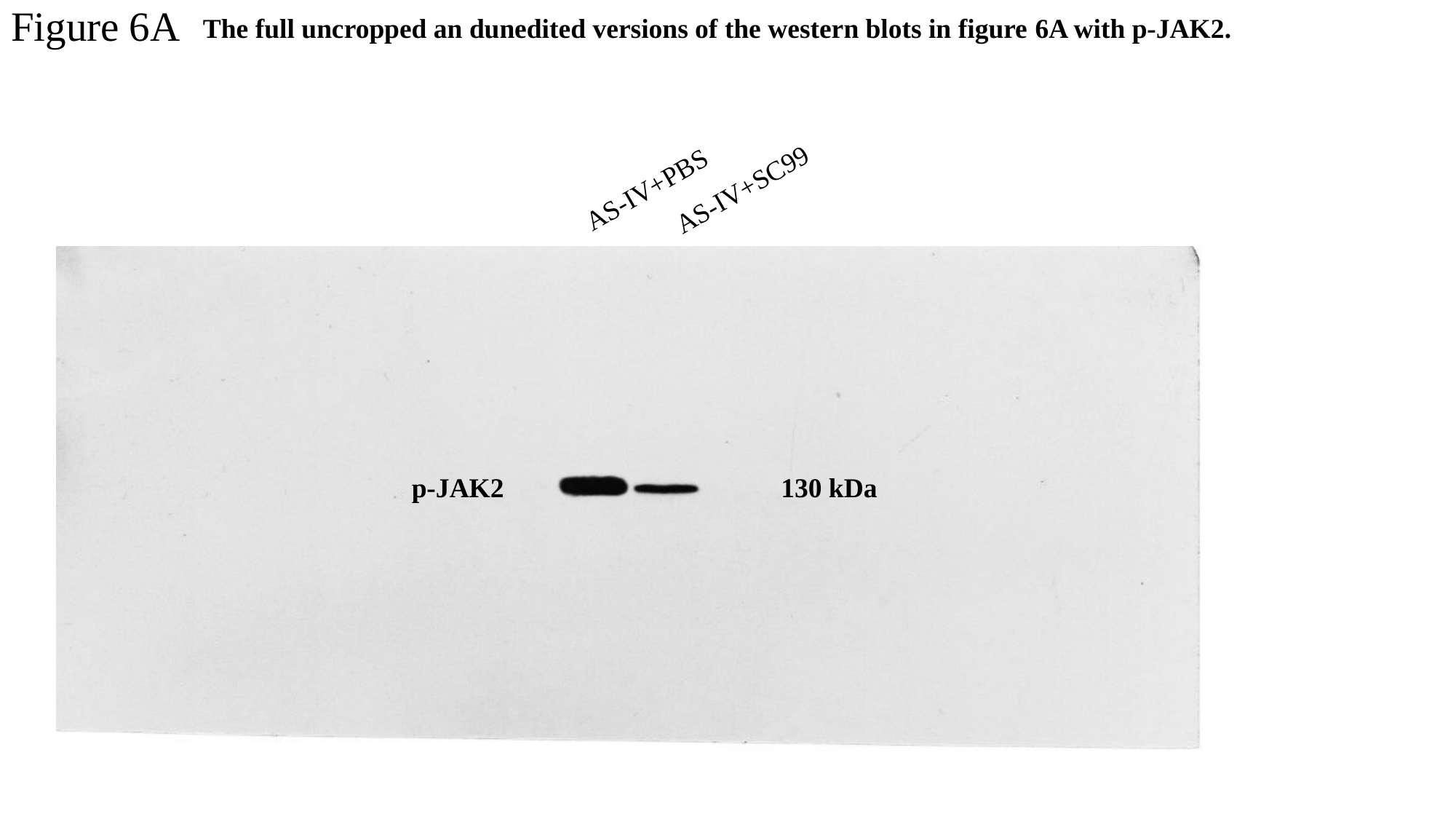

# Figure 6A
The full uncropped an dunedited versions of the western blots in figure 6A with p-JAK2.
AS-IV+SC99
AS-IV+PBS
130 kDa
p-JAK2

## Slide 11
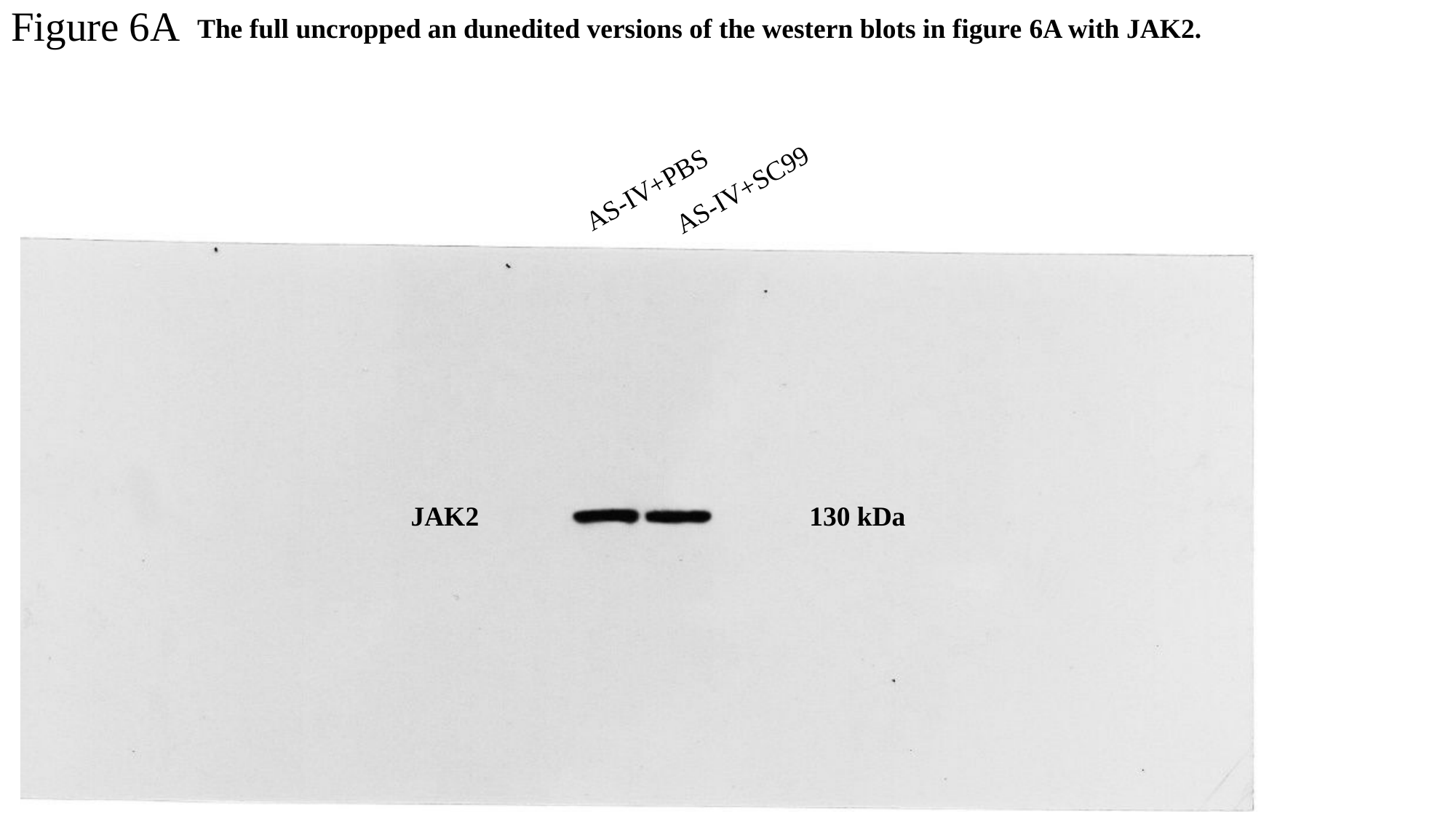

# Figure 6A
The full uncropped an dunedited versions of the western blots in figure 6A with JAK2.
AS-IV+SC99
AS-IV+PBS
130 kDa
JAK2

## Slide 12
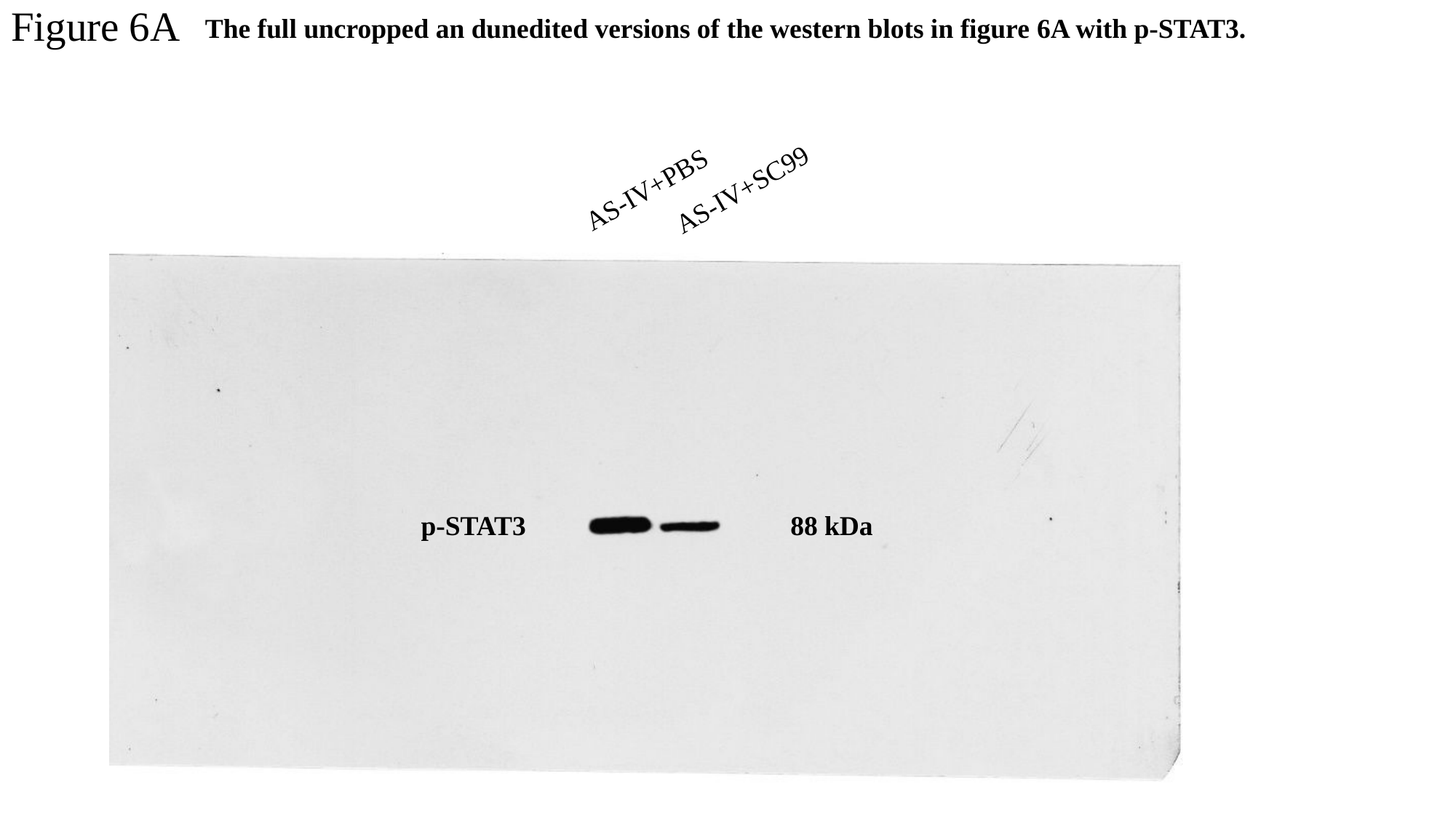

# Figure 6A
The full uncropped an dunedited versions of the western blots in figure 6A with p-STAT3.
AS-IV+SC99
AS-IV+PBS
88 kDa
p-STAT3

## Slide 13
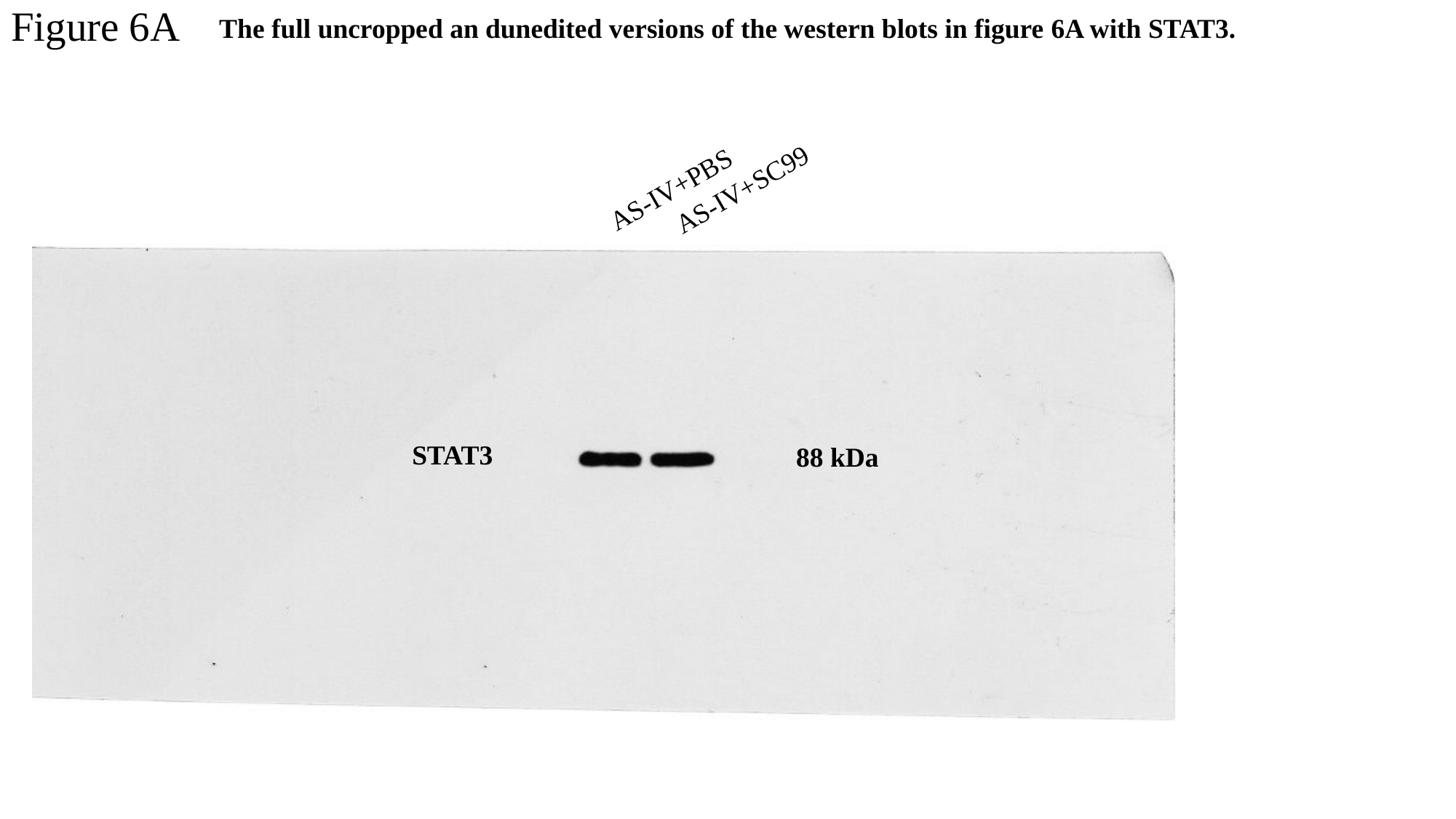

# Figure 6A
The full uncropped an dunedited versions of the western blots in figure 6A with STAT3.
AS-IV+PBS
AS-IV+SC99
STAT3
88 kDa

## Slide 14
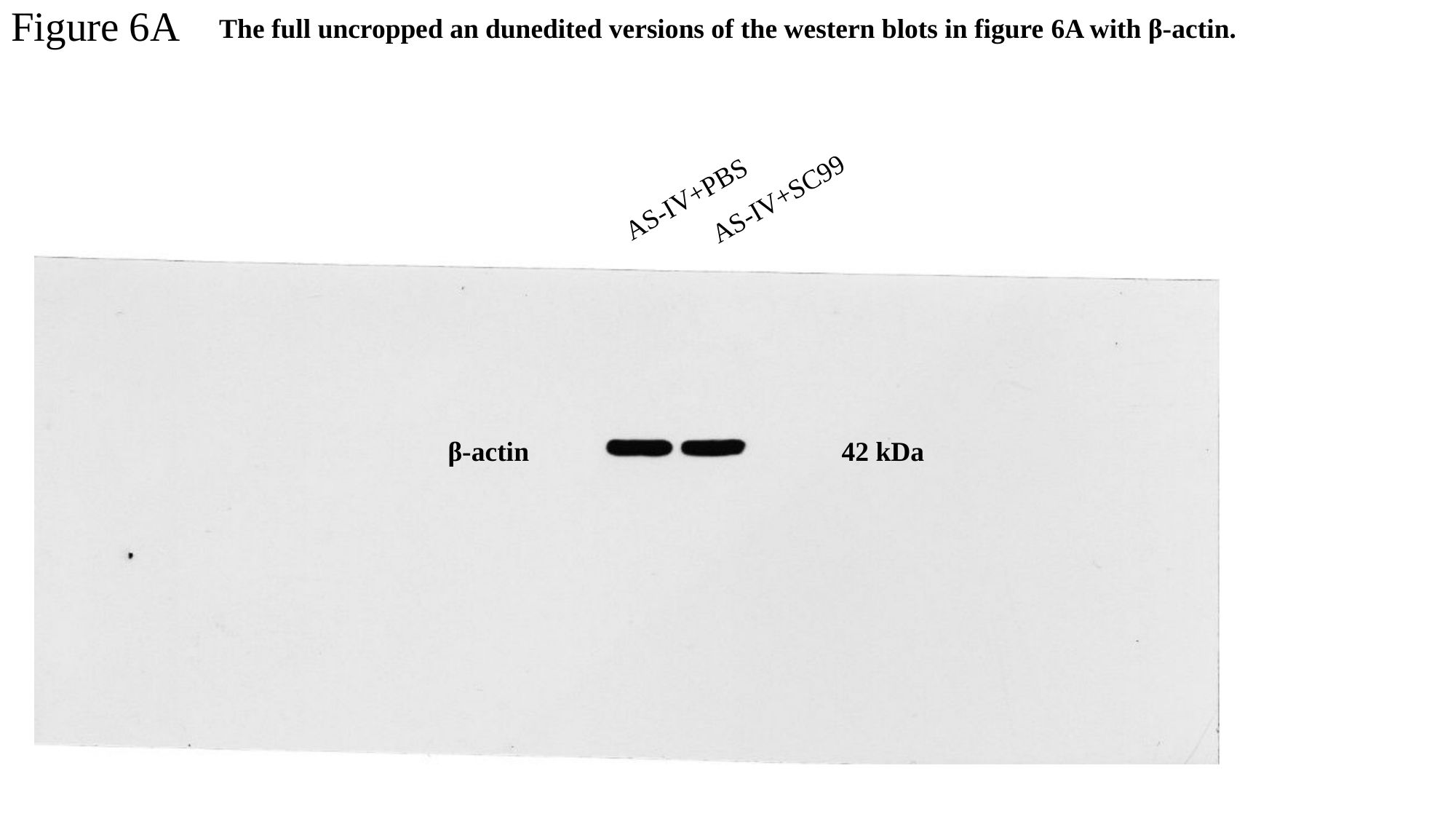

# Figure 6A
The full uncropped an dunedited versions of the western blots in figure 6A with β-actin.
AS-IV+PBS
AS-IV+SC99
42 kDa
β-actin
